# True Hermaphrodite: A Case Report

**Published:** 2011-07-30

**Authors:** Muhammad Zafar Iqbal, Mazhar Rafee Jam, Muhammad Saleem, Mushtaq Ahmad

**Affiliations:** Department of Paediatric Surgery, Sheikh Zayed Medical College Rahim Yar Khan, Pakistan

**Keywords:** True hermaphrodite, Persistent mullerian duct syndrome, Disorders of sexual differentiation

## Abstract

True hermaphrodite is one of the rarest variety of disorders of sexual differentiation (DSD) and represents only 5% cases of all. A 3-year-old child presented with left sided undescended testis and penoscrotal hypospadias. Chordee correction was performed 18 months back, elsewhere. At laparotomy Mullerian structures were present on left side. On right side testis was normally descended into the scrotum.

## INTRODUCTION

True hermaphrodite or ovo-testicular disorder of sexual differentiation (OVO-DSD) is one of the rarest variety of all inter sex anomalies. In about 90% of cases, patients have 46 XX karyotype. Rarely, 46 XY/46 XX mosaicism may occur. There have been reports of 46 XY karyotype [1].

In this condition gonads are asymmetrical having both ovarian and testicular differentiation on either sides separately or combined as ovotestis. In ovotestis, testis is always central and ovary polar in location [2]. Testosterone and Mullerian inhibitory substance (MIS) are either normal or low. However for final diagnosis there must be histological documentation of both types of gonadal epithelium [3]. We are reporting this case for the reason of extreme rarity of this disorder of sexual differentiation with 46 XY Karyotyping.

## CASE REPORT

A 3-year-old patient presented in outpatient department of Sheikh Zayed Hospital Rahim Yar Khan. This child was reared as male and chief complaint was the absence of left testicle in the scrotum along with passage of urine from an abnormal urethral opening at the junction of penis and scrotum. A surgical procedure was performed at 18 months of age to straighten the penis and few injections (testosterone) were given to increase the size of penis, although no record was available.

On examination left testicle was not palpable in the scrotum or inguinal canal. Right testicle was palpable in the scrotum and was of adequate size according to the age of child. Phallus examination showed scar mark on the ventral surface of penis and meatal opening was present at penoscrotal junction. Penis was about 4 cm long with 1.5 cm diameter. All routine investigations were within normal range. Testosterone level was 2.50 micro gram/ dl (normal value 30-50). Barr body test was negative. Karyotyping was 46 XY. Ultrasound report showed left undescended testis which was not visible in inguinal canal or abdomen. No Mullerian structures were noted. A diagnosis of left undescended testis (UDT) with penoscrotal hypospadias was made.

Abdominal exploration for UDT revealed Mullerian structures (ovary, unicornuate uterus, fallopian tube and cervix) on left side (Fig. 1, 2). On Right side vas and vessels were found into deep inguinal ring. After counseling with the parents it was planned to rear this child as male due to predominant male phenotype. The persistent Mullerian structures were excised and sent for histopathology. Biopsy was taken from Right testicle in order to find any dysgenesis or ovotesticular tissue. Biopsy report confirmed ovary, fallopian tube and uterus on left side. Right testicular biopsy showed normal histology with semineferous tubules without any dysgenesis or ovarian tissue.

**Figure:**
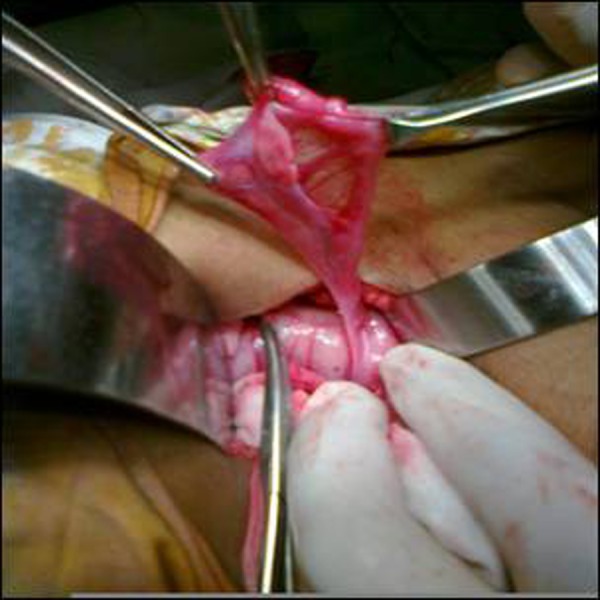
Figure 1: Showing ovary and fallopian tubes.

**Figure:**
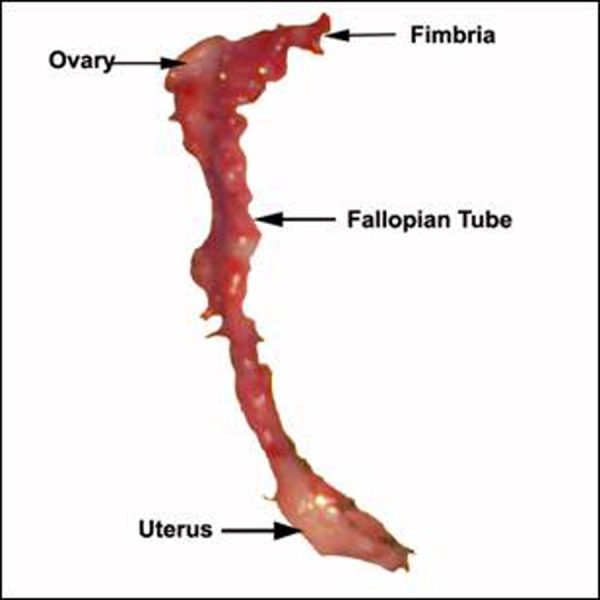
Figure 2: Excised Mullerian remnants.

## DISCUSSION

Disorder of sexual differentiation is the terms used for a child born without clear male or female phenotype. The term “hermaphrodite” is derived from Greek mythological God “Hermaphroditos” son of Hermes and Aphrodite, whose body after being merged with nymph Salmakis assumed a more perfect form with both male and female attributes [4].

Proper gender assignment to a neonate born with DSD is a social emergency of the newborn period. Infants and children born with DSD pose a diagnostic and therapeutic challenge to the clinicians. Success depends upon rapid and precise diagnosis, appropriate gender assignment, proper medical therapy and meticulous surgical technique [5].

The causes of true hermaphroditism remain enigmatic and the commonest presentation is an abnormal external genitalia ranging from normal male to normal female. In many of these cases such distinction may not be present and chordee, hypospadias and cryptorchidism may be noted. Similar picture is found in our case. Other presenting symptoms are hematuria, amenorrhea, lower abdominal pain and inguinal hernia [6, 7, 8].

Documentation of location of gonads is important. In true hermaphrodites gonads are always asymmetrical with predominant testis descends and predominant ovary lies in the abdomen above the external ring as noted in index case. On the basis of location of gonads and histology these patients are classified as: ·Lateral: Testis and contralateral ovary (30%). ·Bilateral: Testicular and ovarian tissue identified on both sides, usually as ovotestis (50 %). ·Unilateral: Ovotestis on one side and testis or ovary on other side (20%). Our patient was of lateral variety in which testis was on right side and ovary on left side. The choice of rearing hermaphrodite as male or female sex is governed by phallus size [9]. In our patient penis was of adequate size thus plan in consultation with parents was made to rear him as a male. All female structures were thus removed. A repair of hypospadias will be performed in the next stage. Prosthesis can be placed in left hemiscrotum for psychological comfort.

True hermaphroditism is rarely associated with gonadal tumours, unlike in mixed gonadal dysgenesis, where the presence of a dysgenetic gonad predisposes to gonadal malignancy. However a few cases of malignancies like dysgerminoma and gonadoblastoma have been reported in true hermaphroditism [10]. Hence this patient will require close follow up to diagnose any malignancy arising in his remaining testis. Since the incidence of gonadal malignancy is low, estimated at 4.6 %, [1] prophylactic removal of his remaining testis is not justified.

Our case was unique as chromosomal analysis was 46 XY, which is very rare in a case of true hermaphrodite DSD.

## Footnotes

**Source of Support:** Nil

**Conflict of Interest:** None declared
